# Impact of Alcalase Hydrolysis and Simulated Gastrointestinal Digestion on the Release of Bioactive Peptides from *Erythrina edulis* (Chachafruto) Proteins

**DOI:** 10.3390/ijms25179290

**Published:** 2024-08-27

**Authors:** Jessica L. Correa, José Edgar Zapata, Blanca Hernández-Ledesma

**Affiliations:** 1 Nutrition and Food Technology Group, Universidad de Antioquia, Medellin 050010, Colombia; jlcorreac@ut.edu.co (J.L.C.); edgar.zapata@udea.edu.co (J.E.Z.); 2 Development and Innovation in Alternative Proteins Group (INNOVAPROT), Institute of Food Science Research (CIAL, CSIC-UAM, CEI UAM + CSIC), Nicolás Cabrera, 28049 Madrid, Spain

**Keywords:** *Erythrina edulis*, enzymatic hydrolysis, in vitro gastrointestinal digestion, peptides, antioxidant, macrophages

## Abstract

Amidst increasing awareness of diet-health relationships, plant-derived bioactive peptides are recognized for their dual nutritional and health benefits. This study investigates bioactive peptides released after Alcalase hydrolysis of protein from chachafruto (*Erythrina edulis*), a nutrient-rich South American leguminous plant, focusing on their behavior during simulated gastrointestinal digestion. Evaluating their ability to scavenge radicals, mitigate oxidative stress, and influence immune response biomarkers, this study underscores the importance of understanding peptide interactions in digestion. The greatest contribution to the antioxidant activity was exerted by the low molecular weight peptides with ORAC values for the <3 kDa fraction of HES, GD-HES, and GID-HES of 0.74 ± 0.03, 0.72 ± 0.004, and 0.56 ± 0.01 (μmol TE/mg protein, respectively). GD-HES and GID-HES exhibited immunomodulatory effects, promoting the release of NO up to 18.52 and 8.58 µM, respectively. The findings of this study highlighted the potential of chachafruto bioactive peptides in functional foods and nutraceuticals, supporting human health through dietary interventions.

## 1. Introduction

The growing awareness of the relationship between diet and health has driven the search for natural sources of beneficial compounds for humans [1,2]. In this context, plant-derived bioactive peptides have emerged as promising compounds that not only contribute to essential nutrition but also offer functional properties and health benefits. These peptides, obtained through the hydrolysis of plant proteins, possess unique structures that confer diverse biological activities [3]. Plants, rich in proteins, represent a valuable reservoir for obtaining peptides with antioxidant, anti-inflammatory, and other health benefits [4,5,6,7]. Because of their acknowledged positive impact on human health, these peptides have become fundamental in the functional foods and nutraceuticals industries. The growing interest of the pharmaceutical and food sectors in plant-derived ingredients is evident, driven by the numerous advantages they offer over animal-derived materials. These advantages extend not only to their intrinsic health-promoting qualities but also to their potential as functional ingredients in various food and pharmaceutical applications [8].

Although many biological properties of peptides have been demonstrated by in vitro studies, their applicability under in vivo physiological conditions is still limited. This is mainly due to the structural instability and effective digestion of bioactive peptides in the gastrointestinal environment, which has hindered their widespread application as nutraceuticals or functional food ingredients [9]. The digestion behavior of peptides plays an essential role in determining their bioavailability and, consequently, their efficacy in exerting physiological effects within the human body. 

Chachafruto (*Erythrina edulis*), an endemic leguminous plant in South America, has reemerged in importance owing to its rich nutritional composition (crude protein: 25.66%, carbohydrates: 68.76%). It is a notable source of dietary fiber, protein, vitamins, carbohydrates, and antioxidants [10,11,12]. In Colombia, its production shows marked seasonality during the months of April to May and October to November; trees that are 3 to 4 years old can produce 25–29 kg of legumes per hectare per year [10]. Recent research on the proteins of *E. edulis* has highlighted their potential as a source of bioactive peptides with various functional properties and health benefits [13,14]. These findings support the importance of studying and better understanding the properties and applications of proteins derived from this leguminous plant.

Enzymatic hydrolysis is recognized as the preferred method for extracting bioactive peptides, offering a means to enhance essential peptide properties such as activity and bioavailability [3]. Peptides from chachafruto exhibit significant promise for various health-promoting effects [15], rendering them a compelling subject for scientific investigation. However, the behavior of these peptides during gastrointestinal digestion has not been studied yet. The intricate crossing of these peptides—from ingestion through absorption—involves complex interactions with digestive enzymes and physiological processes in the gastrointestinal tract. A comprehensive understanding of these dynamics is essential for optimizing the utilization of chachafruto-derived peptides in the development of functional foods and nutraceutical formulations. Therefore, the objective of this study was to explore the capacity of simulated gastrointestinal digestion under physiological conditions to release or preserve peptides with multiple functions from an Alcalase hydrolyzate of chachafruto protein. The emphasis was placed on evaluating their ability to scavenge radicals and provide protection against oxidative stress and biomarkers associated with the immune response.

## 2. Results and Discussion

### 2.1. Enzymatic Hydrolysis of Chachafruto Seed Protein Concentrate (CPC)

The chachafruto protein concentrate (CPC) was subjected to hydrolysis by Alcalase under the optimal conditions for this microbial enzyme [16]. The yield of hydrolyzed CPC was 38.4% (db) with 95.2% purity. The degree of hydrolysis (DH) was measured during the hydrolysis over a 120 min period. Within the initial 20 min, DH notably increased, reaching a value of 19.6%. Subsequently, the rate of increase gradually diminished, reaching a final DH value of 25.7% at the end of the hydrolysis (120 min). Previous studies reported a higher DH (37.03%) for a protein concentrate obtained from the same seed [14]. However, it is noteworthy that these authors determined the DH by measuring free amino groups (H) through reaction with o-phthalaldehyde (OPA). In contrast, our study employed direct titration using the pH-stat method, which is based on the number of protons released during hydrolysis [17]. It has been pointed out that there is a poor correlation between the results obtained by our method and others used to determine DH, such as the trinitrobenzenesulfonic acid (TNBS), OPA, and soluble nitrogen in trichloroacetic acid (SN-TCA) protocols; thus, a direct comparison between studies is usually not feasible [18,19]. Guerra-Almonacid et al. [13] also reported a higher DH (47.7%) for *E. edulis* protein, which could have been favored by the pre-treatment of the sample with ultrasounds and the sequential hydrolysis with Alcalase and Flavourzyme. Sequential hydrolysis has been employed to increase DH and, consequently, to test enzymes with different specificities or binding sites, ensuring a greater diversity of peptides. Serrano Cervantes [11] obtained a DH value of 43.02% when the hydrolysis of chachafruto protein was conducted sequentially with pepsin, pancreatin, and Alcalase. The applicability of the hydrolyzate can be determined by the DH. Hydrolyzates with a DH below 10% (limited) are generally used to enhance functional properties such as solubility, foaming capacity, and emulsifying ability in certain foods. On the other hand, hydrolyzates with DH above 10% (extensive) are usually employed in pharmaceutical formulations (nutraceuticals) or functional foods due to the bioactive properties of released peptides [20].

### 2.2. Characterization of Chachafruto Protein Concentrate Hydrolyzate (HES)

**Figure 1:**
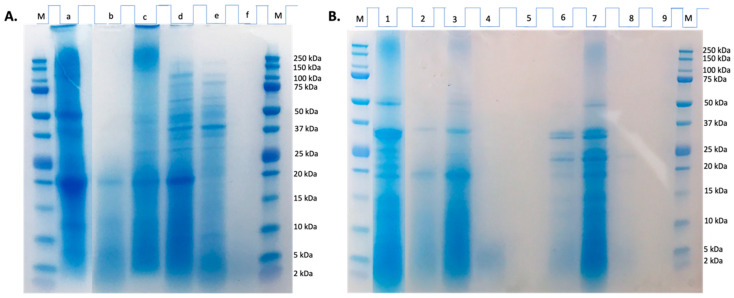
Electrophoretic (SDS-PAGE) analysis of *Erythrina edulis* (chachafruto) seed protein concentrate (CPC) hydrolyzate (HES) before and after its digestion, simulating gastrointestinal conditions, and fractions collected from undigested and digested HES by ultrafiltration. (**A**) HES before gastrointestinal digestion. M: molecular marker; a: CPC; b: HES; c: Protein fraction > 100 kDa; d: peptidic fraction 10–100 kDa; e: peptidic fraction 3–10 kDa; f: peptidic fraction ˂ 3 kDa. (**B**) HES after gastrointestinal digestion. M: molecular marker; 1: digestion blank; 2: gastric digest from HES (GD-HES); 3: peptide fraction > 10 kDa from GD-HES; 4: peptide fraction 3–10 kDa from GD-HES; 5: peptide fraction < 3 kDa from GD-HES; 6: gastrointestinal digest from HES (GID-HES); 7: peptide fraction > 10 kDa from GID-HES; 8: peptide fraction 3–10 kDa from GID-HES; 9: peptide fraction < 3 kDa from GID-HES. The amount of protein loaded onto each well was 50 µg protein.

As illustrated in Figure 1, the electrophoretic profile of the CPC (lane a) revealed bands ranging between 5 and 250 kDa (Figure 1A), being the most intense; those ranged from 20 to 50 kDa. CPC showed a typical profile from legume protein concentrates, with bands of high molecular mass (above 75 kDa, 100, and 250 kDa) that could be attributed to globulin fractions and polypeptides of intermediate (50, 37, and 25 kDa) and low molecular mass (≤20 kDa) that correspond to globulin and albumin fractions [21]. Thus, the presence of major bands in the range of 25 and 50 kDa might correspond to the basic and acidic subunits of 11S globulin. One of the most intense bands observed for the CPC, corresponding to aprox. 50 kDa, could be associated with vicilin (48 kDa) [22]. This result was consistent with our previous study [23]. After Alcalase hydrolysis, the HES profile (lane b) exhibited bands within the 5–20 kDa range, indicating that chachafruto proteins were partially susceptible to the action of this enzyme, releasing low-molecular-weight proteins and peptides. The ultrafiltered fractions from HES exhibited a wide variety of bands. No bands were observed in the line corresponding to the peptide fraction ˂3 kDa (lane f), indicating that after proper ultrafiltration, small peptides contained in this fraction were not detected because of the electrophoretic conditions used. However, the electrophoretic profiles of lanes e and d were very similar (Figure 1A), which could be due to an inadequate ultrafiltration process that allowed proteins from the 10–100 kDa fraction to pass into the 3–10 kDa fraction.

The amino acid composition of HES, expressed as g/100 g protein and g/100 g HES, is detailed in Table 1. Seventeen amino acids were detected in the HES, except tryptophan (Trp), which was not detected because of its degradation during acid hydrolysis. Leucine (Leu) and tyrosine (Tyr) were the most abundant essential amino acids (EAA) with values of 6.10 ± 0.20 and 3.77 ± 0.15 g/100 g protein, respectively; on the other hand, among the non-essential amino acids (NEAA), the most abundant were glutamic acid + glutamine and aspartic acid + asparagine, with values of 9.48 ± 0.35 and 7.49 ± 0.29 g/100 g of protein, respectively. The EAA/total AA (TAA) ratio is an index of good-quality proteins [24]. The recommended protein standard announced by the Food and Agriculture Organization of the United Nations/World Health Organization (FAO/WHO) in 1973 suggested that this ratio of EAA/TAA should be 40% and the ratio of EAA/NEAA should exceed 60% [25]. The amino acid composition for HES revealed EAA/TAA and EAA/NEAA ratios of 42.0% and 72.5%, respectively, higher than the recommended protein standard (Table 1). It has been reported that hydrophobic amino acids (HAAs) enhance the potency of antioxidant peptides by serving as proton and electron donors and direct scavengers of lipid radicals, while aromatic amino acids (AAAs) such as Trp and Tyr are amino acids with high antioxidant activity due to their indolic and phenolic groups, which act as hydrogen donors binding to oxygen radicals, forming more stable compounds [26,27]. The amount of HAA and AAA present in the HES was significant, with values of 44.3% and 12.2% of TAA, respectively, making HES a potential source of antioxidant peptides.

### 2.3. Behavior of Chachafruto Protein Concentrate Hydrolysate (HES) under Simulated Gastrointestinal Digestion

HES was subjected to gastrointestinal digestion, simulating physiological conditions. The fractions obtained by ultrafiltration showed yields for HES of 16.01% (F > 10 kDa), 13.65% (F 3–10 kDa), and 23.27% (F < 3 kDa); for GD-HES of 12.57% (F > 10 kDa), 9.44% (F 3–10 kDa), and 18.41% (F < 3 kDa); for GID-HES of 13.60% (F > 10 kDa), 5.38% (F 3–10 kDa), and 12.15% (F < 3 kDa). The behavior of proteins and peptides contained in HES before and after the action of gastric enzymes was evaluated by SDS-PAGE (Figure 1A,B). When HES was treated with gastrointestinal enzymes (Figure 1B), the corresponding bands for pancreatin (23.8, 38, and 45 kDa for protease, amylase, and lipase, respectively) and pepsin (34.5 kDa) [28,29] were observed in the digestion blank (lane 1), the digested GD-HES and GID-HES (lanes 2 and 6), and its larger fractions (lanes 3 and 7). In the medium and low molecular weight fractions (lanes 4, 5, 8, and 9), no bands were observed, indicating that the filtration process was correctly carried out, preventing the digestive enzymes from passing into these fractions. The absence of bands in these lines also indicated that the action of digestive enzymes during gastric and gastrointestinal digestion of HES provoked the release of small peptides that migrated beyond the gel with a molecular weight below the detection limit.

**Figure 2:**
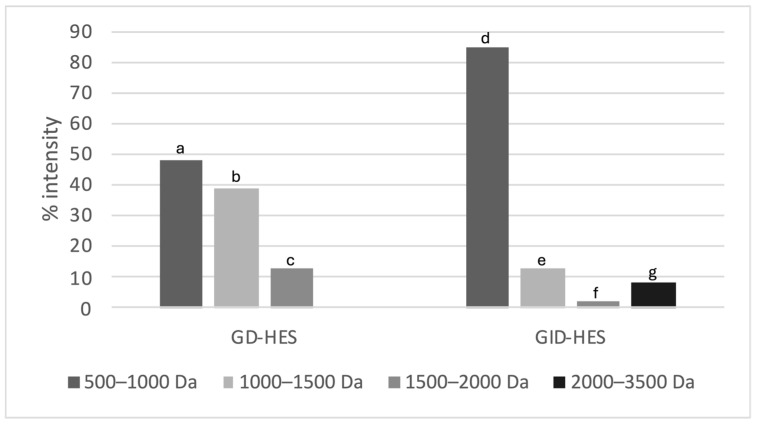
Peptide mass distribution (% intensity) in the gastric and gastrointestinal digests from *Erythrina edulis* (chachafruto) seed protein concentrate hydrolyzate (HES) (GD-HES, GID-HES). Different superscript letters indicated statistical differences.

To confirm the proteolytic action of digestive enzymes on HES, a MALDI-TOF analysis was conducted. Figure 2 shows the distribution of molecular weights of peptides present in GD-HES and GID-HES. In both digests, peptides with molecular weights ranging from 500 to 1000 Da were the most abundant, representing 48.0% and 85.5% of the total identified peptides in GD-HES and GID-HES, respectively. No peptides ranging from 2000 to 3500 Da were identified in the GD-HES, indicating that during gastric digestion, only small peptides were released from proteins and peptides contained in HES, maintaining intact proteins with molecular weights higher than 3500 Da that were not detected with the assay conditions, while the combined action of gastric and pancreatic enzymes allowed the breakage of those proteins, releasing high-molecular-weight peptides (2000–3500 Da) that represented 8.7% of the total identified peptides.

### 2.4. Effect of Simulated Gastrointestinal Digestion on the Antioxidant Activity of Chachafruto Protein Hydrolyzate (HES)

Using biochemical assays, the effect of simulated gastrointestinal digestion of HES on its antioxidant activity and the influence of the molecular weight of the released peptides on this activity were evaluated. The ABTS^•+^ and peroxyl (ROO^•^) radical scavenging activity of HES, GD-HES, and GID-HES and their ultrafiltered fractions were analyzed, and the results are shown in Table 2.

While the ABTS assay is based on the electron transfer mechanism, the ORAC assay is based on the hydrogen atom transfer mechanism. The ABTS radical scavenging activity shown by HES was moderate, with a TEAC value of 0.64 ± 0.04 µmol TE/mg protein. However, this hydrolyzate showed a potent ability to scavenge peroxyl radicals (ORAC value of 1.95 ± 0.11 µmol TE/mg protein). As shown in Table 2, the action of pepsin during the gastric phase resulted in a significant increase in antioxidant activity, reaching TEAC and ORAC values for GD-HES of 0.73 ± 0.01 and 2.72 ± 0.22 µmol TE/mg protein, respectively. However, the effect of pancreatic enzymes on antioxidant activity differed depending on the assay used. Thus, while the TEAC value of GID-HES decreased with respect to that determined for GD-HES, the peroxyl radical chelating capacity continued increasing during the intestinal phase until reaching an ORAC value of 2.96 ± 0.07 µmol TE/mg protein. The release of peptides with radical scavenging capacity from various food sources through sequential enzymatic hydrolysis and simulated gastrointestinal digestion has been previously reported. Vásquez et al. [30] subjected the Alcalase hydrolyzate from rainbow trout viscera to simulated gastrointestinal digestion and observed an increase in its antioxidant activity from ABTS and ORAC values of 1347 and 1395 μmol TE/g, respectively, for the hydrolyzate, to values of 1608 and 1464 μmol TE/g, respectively, for the gastrointestinal digest. The results demonstrated that the action of digestive enzymes allowed the release of new peptides from protein hydrolyzate capable of neutralizing radicals. However, no significant differences in the antioxidant activity were observed when a soy hydrolyzate with Alcalase was subjected to simulated digestion with pepsin and pancreatin, indicating a possible resistance of peptides to the action of digestive enzymes [31]. In our previous study, the gastrointestinal digest from CPC exhibited lower antioxidant activity than that demonstrated in the present study for the digested hydrolyzate [23]. Thus, the reported ABTS and ORAC values for the digested protein were 0.46 and 1.12 µmol TE/mg protein, respectively, whereas values determined in our current study for digested protein hydrolyzates were 0.68 and 2.96 µmol TE/mg protein. These results suggested that previous hydrolysis of chachafruto protein with Alcalase improved the release of antioxidant peptides during simulated gastrointestinal digestion. Controversial results among studies suggest that the biological properties of protein hydrolyzates can be enhanced or diminished after the action of digestive enzymes, depending mainly on the initial protein source and the conditions of the hydrolysis and digestion processes [32]. Thus, understanding how bioactive peptides behave during digestion could provide valuable information on their effects on living organisms and offer a preliminary assessment before resorting to costly animal and human trials [31]. 

For both HES and its digests, the greatest contribution to antioxidant activity was exerted by the low-molecular-weight peptides. Thus, the TEAC and ORAC values determined in the ˂3 kDa fractions from HES, GDHES, and GIDHES were similar or even higher than those determined in the whole sample (Table 2). Peptides with low molecular weight obtained from other food proteins have also demonstrated potent antioxidant activity [33]. Consistent with our findings, earlier research has indicated that Alcalase hydrolysis of different legume proteins resulted in the release of small peptides exhibiting higher radical scavenging activity than that exhibited by long peptides [34]. However, it has been reported that large and medium fractions of hydrolyzates could also contribute to their antioxidant activity. In our study, large and medium peptides contributed similarly to the antioxidant activity measured by both methods. Previous studies have reported that not only the size of peptides is crucial to antioxidant activity, but it also depends on the sequence in which they are arranged and the global composition of the peptide. Hydrophobicity is vital for bioactive peptides to show their antioxidant activity, as they interact with lipid systems both in our body and in food [35]. As previously indicated, HES contained a great percentage of HAA and AAA that could be partially responsible for the potent antioxidant activity observed for the hydrolyzate and its corresponding digests.

### 2.5. Impact of Simulated Gastrointestinal Digestion on the Modulatory Effects of HES in an Immune Cell Model

**Figure 3:**
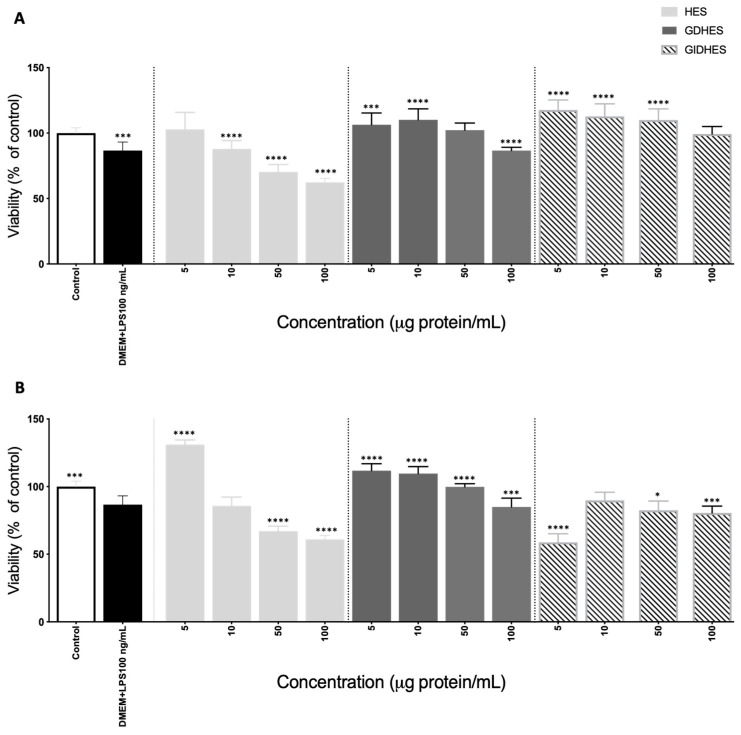
Viability (expressed as % of control considered as 100%) of (**A**) basal RAW264.7 macrophages and (**B**) lipopolysaccharide (LPS)-stimulated RAW264.7 macrophages after 24 h incubation with different concentrations (5–100 μg protein/mL) of undigested and digested chachafruto seed protein concentrate hydrolyzate by Alcalase (HES, GD-HES, and GID-HES). Significant differences compared to the (**A**) control or (**B**) LPS (* *p* < 0.05; *** *p* < 0.001; **** *p* < 0.0001).

To evaluate the antioxidant and immunomodulatory effects of HES and its digests, the RAW264.7 macrophage cell model was utilized under both basal and stimulated conditions. Firstly, the effects of samples on cell viability were evaluated at doses ranging from 5 to 100 μg protein/mL (Figure 3). The 3-[4,5-dimethylthiazol-2-yl]-2,5-diphenyl tetrazolium bromide (MTT) assay revealed, at the lowest dose assayed (5 g protein/mL), no cytotoxic effects on basal macrophages (Figure 3A). At doses above 10 µg protein/mL, HES exerted a dose-dependent cytotoxic action. However, the cytotoxic effects of GD-HES and GID-HES were only observed when cells were treated with doses higher than 100 µg protein/mL, indicating the toxic protein compounds present in HES were degraded by the action of digestive enzymes. Our previous study also reported a reduction in the cytotoxic effects of CPC after its gastrointestinal digestion [23]. As shown in Figure 3A, GD-HES (5–10 μg protein/mL) and GID-HES (5–50 μg protein/mL) exerted an immunostimulatory action favoring macrophage viability. Under lipopolysaccharide (LPS, 100 ng/mL) challenge, cell viability decreased up to 86.62 ± 6.53%. However, the pre-treatment of cells with low doses of HES and GD-HES reverted the cytotoxic effect of LPS (Figure 3B). Even so, they promoted the proliferation of macrophages at concentrations of 5 μg protein/mL (HES) and 5–50 μg protein/mL (GD-HES), confirming the immunostimulatory action of the proteins/peptides contained in these samples. However, HES at doses higher than 50 μg protein/mL potentiated the cytotoxic action of LPS, as was also found for GID-HES (Figure 3B). Based on these findings, it was decided to work with concentrations of 50 µg protein/mL for the cell assays, which did not exert any effect on the cell viability.

**Figure 4:**
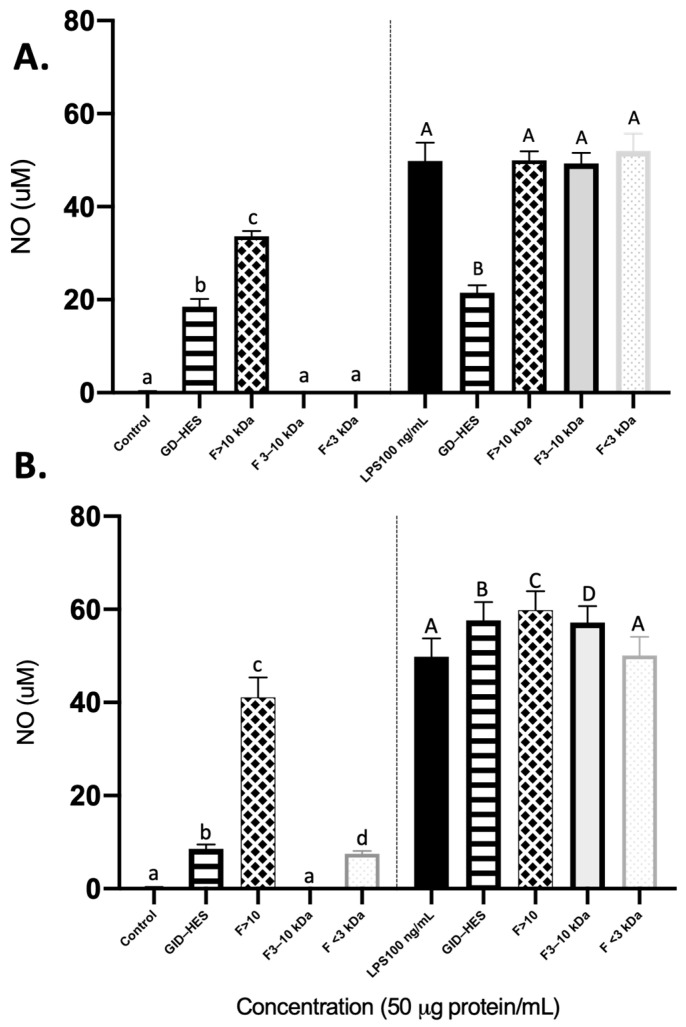
Nitric oxide (NO) production (μM) by basal (**left side**) and LPS-stimulated (**right side**) RAW264.7 after 24 h of exposure to (**A**) Gastric digest from Alcalase hydrolyzate (GD-HES) and its fractions higher than 10 kDa (F > 10 kDa), from 3 to 10 kDa (F 3–10 kDa), and lower than 3 kDa (F < 3 kDa). (**B**) Gastrointestinal digest from Alcalase hydrolyzate (GID-HES) and its fractions higher than 10 kDa (F > 10 kDa), from 3 to 10 kDa (F 3–10 kDa), and lower than 3 kDa (F < 3 kDa). Means with the same letter were not significantly different.

Macrophages undergoing activation have essential functions in both cell-mediated and humoral immunity, and their viability serves as an indicator of the immunomodulatory effects and toxicity of immune activators. Macrophages perform essential functions in the immune system, including phagocytosis to remove necrotic debris at injury sites, nitric oxide (NO) production to combat pathogens, and secretion of various cytokines and enzymes involved in the inflammatory response [36]. In cases of microbial infection or inflammation, activated macrophages release NO, a significant inflammatory mediator, playing a role in host immune defense, tissue repair, and various physiological activities [37]. The released NO in activated macrophages exhibits non-specific cytotoxic effects, not only eliminating invading microorganisms but also inhibiting the proliferation of both cancer cells and tumor cells [38]. Macrophages stimulated by LPS serve as a common in vitro experimental model for assessing the anti-inflammatory properties of natural products [39]. As shown in Figure 4A,B, GD-HES and GID-HES promoted the release of NO up to 18.52 and 8.58 µM, respectively. 

The main reason for this immunostimulatory effect was the fractions higher than 10 kDa obtained from both digests, as they increased NO levels up to 33.64 and 41.13 µM, respectively. In the case of GID-HES, a slight but significant NO-promoting effect was observed when cells were treated with a fraction lower than 3 kDa (7.41 µM). For LPS-challenged cells (right side of Figure 4A), GD-HES reverted the NO-inducing effects caused by LPS, whereas its ultrafiltered fractions did not show any effect. However, GID-HES and its fractions higher than 10 kDa and 3–10 kDa significantly potentiated the effects of LPS (Figure 4B), while fractions lower than 3 kDa did not modify them. It can be concluded that the generation of NO by activated macrophages was substantially suppressed by the peptides contained in the smallest fraction of GID-HES. Various investigations have shown that natural derivatives inhibit the LPS-induced production of NO in RAW 264.7 macrophage cells by suppressing the expression of inducible NO synthase (iNOS) [40]. The digests in contact with LPS-stimulated macrophages showed a marked increase in NO release as compared with the non-stimulated cells. This finding indicates that RAW264.7 macrophages were activated, leading to increased NO production and enhanced innate immunity upon stimulation with HES digests.

**Figure 5:**
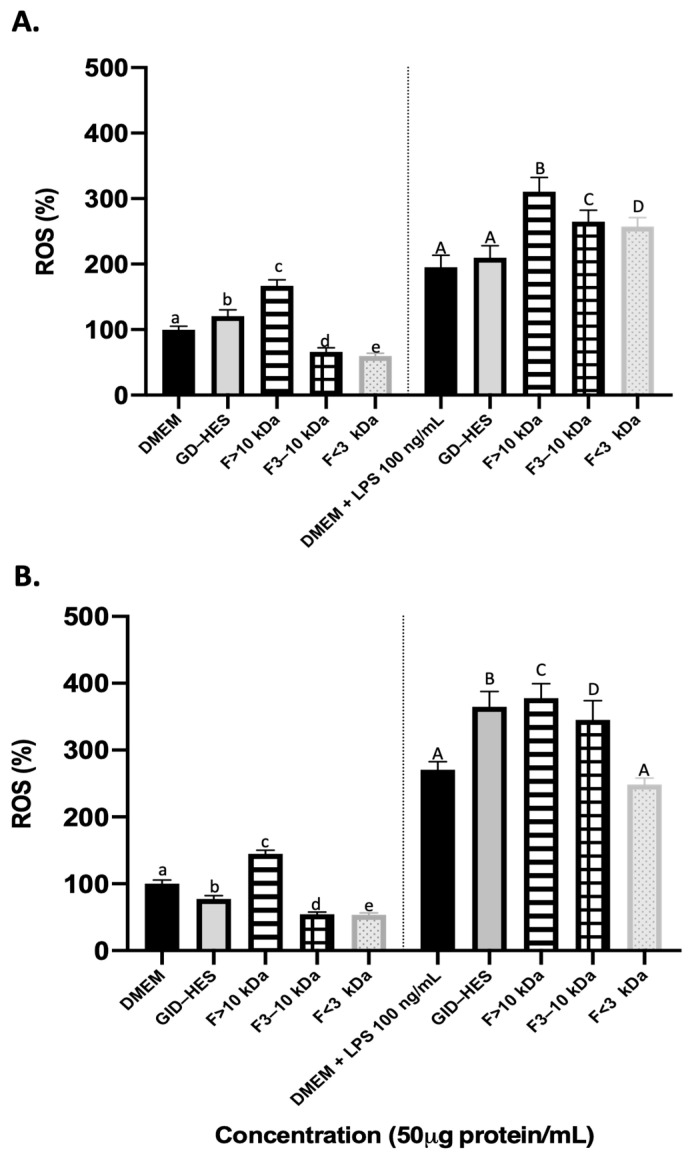
ROS production (%) by basal (**left side**) and LPS-stimulated RAW264.7 (**right side**) after 24 h of exposure to (**A**) Gastric digest (GD) from Alcalase hydrolyzate and its fractions higher than 10 kDa (F > 10 kDa), from 3 to 10 kDa (F 3–10 kDa), and lower than 3 kDa (F < 3 kDa). (**B**) Gastrointestinal digest (GID) from Alcalase hydrolyzate and its fractions higher than 10 kDa (F > 10 kDa), from 3 to 10 kDa (F 3–10 kDa), and lower than 3 kDa (F < 3 kDa). Means with the same letter were not significantly different.

Reactive oxygen species (ROS) are molecules that are biologically produced during cell metabolism and are involved in cell proliferation and survival. However, the accumulation of ROS may be driven by outside stimuli, such as environmental factors, that cause an imbalance in their production and removal from cells antioxidative systems. This may lead to damage to the cells by oxidative stress [41]. Natural peptides have garnered attention as antioxidants because of their notable effectiveness and minimal toxicity [42]. Thus, exploring the potential of natural peptides to ameliorate diseases caused by oxidative damage represents a promising opportunity for further investigation. After food consumption, gastrointestinal enzymes have been found to aid in the production of antioxidant peptides [43,44]. However, there is a lack of studies on the bioactivity of peptides produced from simulated gastrointestinal digestion and the possible effect of prior hydrolysis by proteases. In this study, to assess the impact of peptides derived from GD-HES and GID-HES on cellular oxidative status, the levels of ROS were measured. Figure 5 summarizes the results obtained in the generation of ROS when macrophage cells under basal and stimulated conditions were exposed to GD-HES (Figure 5A) and GID-HES (Figure 5B) and their respective fractions.

Under basal conditions, the generation of ROS was inhibited by medium and smallest fractions from GD-HES (66.21 and 59.61%, respectively), while it was promoted by the whole digest and fraction > 10 kDa (Figure 5A), reaching values of 120.82 and 166.97%, respectively. While the medium and smallest fractions of gastric digest of the chachafruto protein without prior hydrolysis only reached antioxidant values of 82.02 and 70.23% [23], GID-HES and their 3–10 kDa and <3 kDa fractions inhibited ROS production in comparison with the control, while ROS levels increased after the treatment with the fraction > 10 kDa (Figure 5B). For all cases, it was possible to show that ROS production was lower in the gastric and gastrointestinal digests that were previously hydrolyzed with Alcalase, indicating that prior hydrolysis of the concentrate helps the production of antioxidant peptides. 

Stimulation of RAW 264.7 cells with LPS resulted in increased ROS generation that was potentiated by the treatment of both digests and their ultrafiltered fractions, except for fractions lower than 3 kDa from GID-HES that did not exert any significant effect. The antioxidant or pro-oxidant activity of certain peptides and amino acids is determined by specific conditions, such as their concentration and the pH of the medium [45,46]. Understanding the above can provide new insights into the little-known mechanisms that alter metabolism in macrophages, leading to the production of anti-inflammatory mediators [47].

## 3. Materials and Methods

### 3.1. Preparation of Chachafruto Seed Protein Concentrate (CPC)

The CPC was obtained as previously reported [23]. For flour production, seeds were carefully selected and separated from the pod. The selected plant material was soaked for 12 h to remove antinutritional and water-soluble compounds, which also facilitated the removal of the testa. The grains were dried in a forced-air oven (Thermo Scientific™, Waltham, MA, USA) for 60 h at 40 °C until they reached a relatively low moisture content (less than 10%). Subsequently, the dried seeds were ground in a food processor at 25,000 rpm (De’ Longhi Group, Upper Saddle River, NJ, USA) and sieved using a 0.2 mm mesh. After dissolving the seed flour in water (10% *w*/*v*), its pH was adjusted to 10.0, and the suspension was magnetically stirred for 2 h at 60 °C. Once centrifuged using a refrigerated centrifuge U-320R (Boeco, Germany) at 4500× *g* for 20 min at 25 °C, the supernatant was collected, and its pH was adjusted to 4.5, leaving the suspensions at 4 °C overnight. The precipitated proteins were collected by centrifugation at 10,000× *g* for 20 min at 4 °C, resuspended in water, lyophilized, and stored at −20 °C until further analyses.

### 3.2. Enzymatic Hydrolysis CPC

Enzymatic hydrolysis of the CPC was performed with the food-grade microbial protease Alcalase 2.4 L according to the pH-stat method in a 0.5 L reaction vessel (Metrohm Titrando, Herisau, Switzerland). In brief, the jacketed reaction vessel containing the CPC (8 mg protein/mL) mixed with 500 mL of distilled water was heated at 60 °C for 10 min. The pH of the suspension was adjusted to 8.8 using 0.5 N NaOH to achieve optimal enzymatic activity. The protease was added (10% *w*/*w* based on the substrate), and the hydrolysis was conducted for 2 h. During the hydrolysis, the pH and temperature of the mixture were maintained at the desired level (pH 8.8, 64 °C) by adding 0.5 N NaOH. The solution was magnetically stirred. The volume of NaOH used was measured by an auto-titrator, and the data were used to calculate the DH. The hydrolysis reaction was terminated by placing the reaction mixture in a water bath at 95 °C for 10 min to inactivate the enzyme, followed by cooling to room temperature. The supernatant of protein was collected after centrifuging at 4000× *g* for 15 min at 4 °C, and its pH was adjusted to pH 7.0. The CPC hydrolyzate (HES) was lyophilized and stored at −20 °C until use.

### 3.3. Characterization of the Chachafruto Protein Hydrolyzate (HES)

#### 3.3.1. Degree of Hydrolysis (DH)

The DH, expressed as the percent ratio between the number of peptide bonds broken during the hydrolysis (h) and the total number of peptide bonds in the native protein per unit weight (htot), was calculated from the volume and concentration of NaOH added to maintain the pH constant during the hydrolysis, according to [48], Equation (1):

(1) $$DH\left(\%\right)=\frac{h}{htot}=\frac{B\times NB}{MP\times htot\times \alpha }\times 100$$

where B = Volume of NaOH consumed (mL), NB = Normality of NaOH, MP = Mass (g) of protein (N × 6.25), h_tot_ = Total number of peptide bonds in the substrate (mmol/g protein) determined from its amino acid composition, and α = Average degree of dissociation of α-amino groups released during the hydrolysis, expressed as:

(2) $$\alpha =\frac{10^{pH-pK}}{{1+10}^{pH-pK}}$$

pH and pK are the values at which the proteolysis was conducted. The pK value is dependent on temperature, according to Equation (3):

(3) $$pK=7.8+\frac{298-T}{298\times T}\times 2400$$

#### 3.3.2. Amino Acid Content

The analysis of the amino acid composition in HES involved cation exchange chromatography, conducted using a Biochrom 30 series Amino Acid Analyser (Biochrom, Cambridge, MA, USA). Post-column derivatization with ninhydrin and measurement of absorbance at 570 nm were performed automatically. Prior to analysis, the samples underwent hydrolysis with 6 M HCl for 21 h at 110 °C. The results were presented as the mean of two replicates, expressed in g of amino acid per 100 g of protein.

#### 3.3.3. Electrophoretic (SDS-PAGE) of Chachafruto Seed Protein Hydrolyzate (HES)

The protein profiles of HES, their gastrointestinal digests, and ultrafiltered fractions were examined through SDS-PAGE employing 12% polyacrylamide gels (Bis-Tris CriterionTM XT Precast Gel, Bio-Rad, Hercules, CA, USA). The samples were prepared by mixing the samples with a sample buffer [60 mM Tris-HCl pH 6.8, 25% glycerol (*v*/*v*), 2% sodium dodecyl sulfate (SDS) (p/v), 14.4 mM 2-mercaptoethanol, and 0.1% 2-bromophenol (p/v)], followed by heating for 5 min at 100 °C and cooling to room temperature before loading onto gels (50 µg protein/sample). The analysis was conducted using a Criterion automated system (Bio-Rad) with XT MES Running Buffer 20X (Bio-Rad). Electrophoretic migration was carried out at a voltage of 150 V for 45 min. Subsequently, the gels were stained with Coomassie Blue for 60 min and washed with a 10% acetic acid − 10% methanol solution for 12 h. Precision Plus Protein Standard Unstained (Bio-Rad) served as the molecular weight protein standard.

### 3.4. Simulated Gastrointestinal Digestion

The in vitro simulated gastrointestinal digestion of HES was conducted following the INFOGEST harmonized protocol [49] with certain adjustments. To outline, 0.5 g of HES was dissolved in 5 mL of simulated salivary fluid (pH 7.0, 37 °C) for 5 min. Subsequently, 4 mL of simulated gastric juice (pH 3.0, 37 °C) containing porcine gastric mucosa-derived pepsin (2000 U/mL of digest, EC 3.4.23.1, Sigma-Aldrich, St. Louis, MO, USA) was added and incubated at 37 °C for 120 min. Following the adjustment of the pH of gastric digests (GD-HES) to 7.0 with 1 M NaOH, the intestinal phase started with the addition of simulated intestinal fluid comprising porcine pancreas pancreatin (100 U trypsin activity/mL of the final mixture, Sigma-Aldrich) and porcine bile extract (10 mM in the final mixture, Sigma-Aldrich). The digestions (performed in duplicate) took place at 37 °C in an orbital shaker at 150 rpm. After 120 min incubation, the samples were heated at 80 °C for 15 min to inactivate enzymes and obtain the gastrointestinal digest (GID-HES). The samples were then freeze-dried and stored at −20 °C until analysis. A digestion blank (DB), consisting of the enzyme mixture without HES, was prepared.

HES, GD-HES, and GID-HES underwent ultrafiltration through hydrophilic 10,000 and 3000 Da cutoff membranes (Merck KGaA, Darmstadt, Germany). Fractions < 3 kDa, 3–10 kDa, and >10 kDa were lyophilized and stored at −20 °C until use. The bicinchoninic acid method (BCA) (Pierce, Rockford, IL, USA) was employed to measure the peptide content of digests and fractions, using bovine serum albumin (BSA) as the standard protein.

### 3.5. Distribution of Molecular Weight

Peptide mass distribution analysis in GD-HES and GID-HES was conducted using MALDI-TOF mass spectrometry (MS), following a previously described protocol [50]. Samples were applied to an Anchorchip target (Bruker Daltonik GmbH, Bremen, Germany) with α-cyano-4-hydroxycinnamic acid (α-CHCA) matrix in acetonitrile/water (30:70) containing 0.1% trifluoroacetic acid (TFA) and analyzed on a Bruker Autoflex Speed^®^ (Bruker Daltonik). Mass spectra were acquired in positive reflectron mode, averaging 1000 laser pulses. Calibration was performed using Peptide Calibration Standards I and II (Bruker Daltonik).

### 3.6. Antioxidant Activity by Biochemical Assays

The ABTS^•+^ scavenging activity was evaluated using the improved protocol described by [51]. In this procedure, 180 μL of diluted ABTS^•+^ solution was mixed with 20 μL of either PBS (blank), Trolox (25–200 µM) (standard), or the sample (at various concentrations) and incubated for 5 min at room temperature. After incubation, the absorbance was measured at 734 nm using a Synergy HTX microplate reader (BioTek Instruments, Inc., Winooski, VT, USA). The Trolox equivalent antioxidant capacity (TEAC) was determined by dividing the slope of the plot of percentage inhibition of absorbance against the protein concentration of the sample by the slope of the plot for Trolox. The results were expressed as µmol Trolox equivalents (TE) per mg of protein.

The oxygen radical absorbance capacity (ORAC) was determined following the protocol outlined by [52] with some modifications. Specifically, 20 μL of either PBS (blank), Trolox (0.2–1.6 nmol), or the sample (at varying concentrations) was mixed with 120 μL of FL (117 nM) and incubated at 37 °C for 10 min. Subsequently, 60 μL of AAPH (14 mM) was added, and the mixture was incubated for 80 min. Fluorescence readings were taken at excitation and emission wavelengths of 485 nm and 520 nm, respectively, using a FLUOstar OPTIMA plate reader (BMG Labtech, Offenburg, Germany). The ORAC-FL value was calculated as the mean of three replicates and expressed as µmol TE/mg of protein.

### 3.7. Protective Effects in Macrophage RAW264.7 Cells

#### 3.7.1. Cell Culture

The mouse macrophage cell line RAW264.7 (American Type Culture Collection, ATCC, Rockville, MD, USA) was cultured in DMEM medium supplemented with 10% FBS (*v*/*v*) and 1% penicillin/streptomycin (*v*/*v*). Cells were seeded in 75 cm^2^ culture flasks and maintained at 37 °C in a humidified incubator with 5% CO_2_ and 95% air. The culture medium was refreshed every 2 days, and macrophage subcultures were initiated by scraping.

#### 3.7.2. Effects on Cell Viability

Cell viability was assessed using the MTT assay. RAW264.7 cells were seeded onto 96-well plates at a density of 6 × 10^4^ cells/well in complete medium with 10% FBS and incubated for 24 h at 37 °C. Following the removal of the culture medium, samples were added at different concentrations (5, 10, 50, and 100 μg protein/mL). In the case of LPS-stimulated cells, 20 μL of LPS (100 ng/mL, final concentration) was also added. After a further 24 h of incubation at 37 °C, the supernatant was aspirated, and cells were washed with PBS. Subsequently, a MTT solution (5 mg/mL in PBS) was added, and the plate was incubated for 120 min at 37 °C. Upon aspirating the supernatant, insoluble formazan crystals were dissolved in dimethyl sulfoxide (DMSO), and the absorbance was measured at 570 nm using the Multiskan FC plate reader (Thermo^TM^ Scientific, Wilmington, DE, USA). The results were expressed as a percentage of the control, which was considered 100%.

#### 3.7.3. Effects on Reactive Oxygen Species (ROS) Generation

Intracellular ROS levels were quantified following the method outlined by [53], utilizing dichlorofluorescein (DCFH-DA) as a probe. RAW264.7 macrophages were seeded onto 48-well plates (density of 4.75 × 10^4^ cells/well) in complete medium with 10% FBS and incubated for 24 h at 37 °C. Upon aspiration of the medium, samples were added (50 g protein/mL), and cells were incubated for an additional 24 h at 37 °C. For stimulated macrophages, 20 μL of LPS (100 ng/mL) was also added. Following the removal of the supernatant, 100 µL of a solution containing 5 mM DCFH-DA dissolved in Hank’s balanced salt solution (HBSS, Sigma-Aldrich) was added to the wells, and the plate was incubated at 37 °C for 60 min. Fluorescence was measured in a FLUOstar OPTIMA plate reader (BMG Labtech) at an excitation wavelength (λexcitation) of 485 nm and an emission wavelength (λemission) of 530 nm. The results were expressed as ROS levels (% compared with the control, considered as 100%).

#### 3.7.4. Effects on Nitric Oxide (NO) Levels

RAW264.7 macrophages were seeded onto 96-well plates (1 × 10^5^ cells/well) in complete medium with 10% FBS and incubated for 24 h at 37 °C. Subsequently, the medium was aspirated, 100 μL of samples were added (50 g protein/mL), and cells were further incubated for 24 h at 37 °C. For stimulated cells, 20 μL of LPS (100 ng/mL) was added. The accumulation of nitrite, an indicator of NO synthesis, was assessed in the macrophage culture medium using the Griess reaction, following a method previously described [54]. In brief, a mixture comprising 100 μL of supernatant and 100 μL of Griess reagent [1% (*w*/*v*) sulfanyl amide and 0.1% (*w*/*v*) N-1-(naphthyl) ethylenediamine-di-HCl in 2.5% (*v*/*v*) H_3_PO_4_] was incubated for 15 min, and absorbance was measured at 550 nm using a Synergy HTX microplate reader (BioTek Instruments, Inc.). A sodium nitrite standard curve (3.125–100 μM) was employed to quantify the amount of NO. Three independent experiments were conducted, and the data were expressed as the mean and standard deviation (SD) (n = 12).

### 3.8. Statistical Analysis

All data were analyzed in three independent experiments, and the results were expressed as the mean ± standard deviation (SD). Data were analyzed using one-way analysis of variance (ANOVA). All analyses were run with the program GraphPad Prism v.9.0.1 (GraphPad Software, San Diego, CA, USA).

## 4. Conclusions

In this study, CPC was hydrolyzed using Alcalase and digested, simulating gastrointestinal digestion. Electrophoretic analysis revealed that the digestion released low-molecular-weight peptides with potent radical scavenging capacity and the ability to enhance macrophage viability, modulate NO production, and influence ROS levels, indicating potential benefits in both basal and stimulated immune conditions. The findings suggest that pre-hydrolysis with Alcalase improves the bioactivity of CPC during digestion, offering promising applications as an ingredient in new nutraceuticals and/or functional foods directed to prevent/manage diseases associated with oxidative stress and inflammation.

## Figures and Tables

**Table 1:** Amino acid composition (g/100 g protein and g/100 g product) of *Erythrina edulis* protein concentrate (CPC) hydrolyzate (HES).

Amino Acid	Content (g/100 g Protein)	Content (g/100 g Product)	FAO Recommendations
Essential			
Lys	2.99 ± 0.08	2.18 ± 0.06	5.20
Trp	n.d.	n.d.	0.70
Phe	3.56 ± 0.09	2.59 ± 0.06	4.60 ^a^
Tyr	3.77 ± 0.15	2.75 ± 0.11	
Met	0.82 ± 0.03	0.60 ± 0.02	2.60 ^b^
Cys	0.64 ± 0.10	0.47 ± 0.08	
Thr	2.21 ± 0.14	1.61 ± 0.10	2.70
Leu	6.10 ± 0.20	4.45 ± 0.15	6.30
Ile	2.15 ± 0.09	1.57 ± 0.07	3.10
Val	2.96 ± 0.10	2.16 ± 0.07	4.20
Non-essential			
Asx ^a^	7.49 ± 0.29	5.46 ± 0.21	
Glx ^b^	9.48 ± 0.35	6.91 ± 0.25	
Ser	3.85 ± 0.17	2.81 ± 0.12	
His	1.35 ± 0.03	0.98 ± 0.03	
Arg	3.06 ± 0.08	2.23 ± 0.06	
Ala	2.77 ± 0.07	2.02 ± 0.05	
Pro	3.80 ± 0.06	2.77 ± 0.04	
Gly	2.98 ± 0.11	2.17 ± 0.08	
EAA	25.20	18.38	
NEAA	34.78	25.35	
TAA	59.98	43.73	
EAA × 100/TAA (%)	42.0		
EAA × 100/NEAA (%)	72.5		
HAA	26.57	19.37	
AAA	7.32	5.34	
HAA × 100/TAA (%)	44.3		
AAA × 100/TAA (%)	12.2		

n.d., not determined; ^a^ Asp + Asn; ^b^ Glu + Gln. HAA: hydrophobic amino acids (Ala, Val, Ile, Leu, Tyr, Phe, Trp, Met, Prol, and Cys). TAA: total amino acids. AAA: aromatic amino acids (Phe, Trp, and Tyr). Data are the means of two determinations.

**Table 2:** Antioxidant activity (TEAC and ORAC values expressed as µmol TE/mg protein) of enzymatic hydrolysate of protein from *Erythrina edulis* seeds gastric (GD-HES) and gastrointestinal (GID-HES) and their ultrafiltrates.

SAMPLE	TEAC (μmol TE/mg Protein)				ORAC (μmol TE/mg Protein)			
	Whole Sample	F > 10 kDa	F 3–10 kDa	F < 3 kDa	Whole Sample	F > 10 kDa	F 3–10 kDa	F < 3 kDa
HES	0.64 ± 0.04 ^a^	0.42 ± 0.01 ^b,e^	0.43 ± 0.02 ^c,e^	0.74 ± 0.03 ^d^	1.95 ± 0.11 ^a^	1.31 ± 0.02 ^b^	1.48 ± 0.01 ^c^	3.08 ± 0.10 ^d^
GD-HES	0.73 ± 0.01 ^a^	0.39 ± 0.001 ^b,d^	0.37 ± 0.004 ^c,d^	0.72 ± 0.004 ^a^	2.72 ± 0.22 ^a^	1.02 ± 0.09 ^b^	1.22 ± 0.07 ^c^	2.27 ± 0.02 ^d^
GID-HES	0.68 ± 0.01 ^a^	0.33 ± 0.004 ^b,d^	0.34 ± 0.01 ^c,d^	0.56 ± 0.01 ^a^	2.96 ± 0.07 ^a^	1.23 ± 0.08 ^b^	1.17 ± 0.01 ^c^	2.46 ± 0.11 ^d^

TEAC: trolox equivalent antioxidant capacity; TE: trolox equivalent; ORAC: oxygen radical antioxidant capacity. Different superscript letters in the same group indicated statistical differences.

## Data Availability

The original contributions presented in the study are included in the article, further inquiries can be directed to the corresponding author.
